# Phosphorylated Chitosan Modulates the Surface, Mechanical, Optical, and Antibacterial Behavior of Short- and Long-Term 3D-Printed Dental Resins

**DOI:** 10.3390/polym18131576

**Published:** 2026-06-24

**Authors:** Sofia Garibaldi Otavio, Renan Leonardi de Oliveira Rigotti, Tatiane Cristina Dotta, Rogério Valentim Gelamo, Ana Paula Ramos, Rodrigo Galo

**Affiliations:** 1 Department of Dental Materials and Prosthodontics, Ribeirão Preto School of Dentistry, University of São Paulo, Ribeirão Preto 14040-904, SP, Brazil; sofia.garibaldi@usp.br (S.G.O.); renanrigotti@usp.br (R.L.d.O.R.); 2 Department of Chemistry, Ribeirão Preto School of Philosophy, Sciences and Letters, University of São Paulo, Ribeirão Preto 14040-901, SP, Brazil; tatianedotta@usp.br (T.C.D.); anapr@ffclrp.usp.br (A.P.R.); 3 Department of Applied Physics, Federal University of Triângulo Mineiro, Uberaba 38064-200, MG, Brazil; rogerio.gelamo@uftm.edu.br

**Keywords:** three-dimensional printing, chitosan, phosphorylation, incorporation, short-term resins, long-term resins

## Abstract

The incorporation of antimicrobial agents into 3D-printed resins may improve their biological performance; however, their effects on physicochemical and mechanical properties remain unclear. This study evaluated the influence of phosphorylated chitosan (P-Chi; 0.25% and 0.50% *w*/*w*) incorporated into short- (ST) and long-term (LT) 3D-printed dental resins. Surface, mechanical, optical, and antibacterial properties against *Streptococcus mutans* were investigated using standardized methods. FTIR confirmed the successful phosphorylation and incorporation of P-Chi into both resin matrices. P-Chi significantly reduced *S. mutans* CFU counts compared with the control (*p* < 0.001, η^2^p = 0.286), regardless of concentration, although no inhibition halos were detected, indicating a contact-dependent antimicrobial mechanism. Enhanced antibacterial activity was accompanied by increased surface roughness and wettability. Nanoparticle concentration significantly affected mechanical performance (*p* = 0.001), whereas resin type did not (*p* = 0.613). The 0.25% groups exhibited lower flexural strength and microhardness than the controls (*p* < 0.05), while the 0.50% groups maintained flexural strength comparable to that of the controls, with G6 showing the highest elastic modulus (3494.95 ± 301.30 MPa). Color variation was influenced by resin type rather than P-Chi concentration (*p* < 0.05). Overall, P-Chi enhanced antibacterial activity while maintaining clinically acceptable mechanical properties, supporting its use as a multifunctional additive for biofunctional 3D-printed provisional resins.

## 1. Introduction

Additive manufacturing (AM), commonly referred to as 3D printing, enables the fabrication of three-dimensional objects directly from computer-aided design (CAD) data through a layer-by-layer process [1,2], with vat photopolymerization being the dominant approach in dentistry [3,4,5]. This process relies primarily on photopolymer resins, which are broadly classified as short-term (ST) and long-term (LT) formulations [6]. While ST resins exhibit favorable mechanical properties [7,8], LT formulations incorporate higher filler contents to improve marginal adaptation at lower manufacturing costs; nevertheless, both still present mechanical and optical properties inferior to those of CAD/CAM-milled composites and ceramics [9,10,11]. Notably, a 3-year prospective study reported survival rates of only 36.2–40.7% for 3D-printed fixed dental prostheses, with failures predominantly attributed to mechanical complications [11]. In both categories, bacterial colonization is strongly material-dependent and is influenced by surface roughness, composition, and chemistry, which may promote cariogenic biofilm accumulation—particularly by *Streptococcus mutans*—and compromise esthetic and functional predictability [12,13,14].

Therefore, the development of materials with enhanced physical and mechanical properties and reduced bacterial adhesion has been widely explored to optimize the clinical performance of 3D-printed resins. Incorporating particles such as metals, fibers, and oxides, such as aluminum, zirconia, and titanium, into 3D printing resins has emerged as a promising approach to improve their physical, mechanical, and biological properties, ultimately improving clinical performance [15,16,17].

In this context, chitosan (CS), the second-most abundant natural polymer on Earth [18], is obtained by chitin deacetylation and exhibits biocompatibility [19], biodegradability, and intrinsic antibacterial activity, including the ability to reduce bacterial adhesion and biofilm formation [20,21,22,23,24]. Its incorporation into dental materials such as resins [25], cements, denture bases, and other materials have been shown to reduce *S. mutans* adhesion [26] and biofilm metabolic activity while maintaining or improving flexural strength and hardness, particularly at concentrations of 0.25–1 wt% [27,28,29].

Despite these advantages, incorporating CS into hydrophobic resin matrices remains challenging due to its hydrophilic nature, limited solubility in most organic solvents, and poor miscibility with photopolymerizable monomers [30,31], which often result in phase separation, weak interfacial adhesion, and compromised mechanical properties [27,32]. To address these limitations, chemical modification strategies have been proposed, including methacrylated derivatives and copolymerization with monomers such as glycidyl methacrylate (GMA) and poly(ethylene glycol) diacrylate (PEGDA) [33,34]. These approaches primarily aim to improve the compatibility between CS and resin matrices by promoting its participation in the photopolymerization process, leading to enhanced polymer network formation, reduced water sorption and solubility, preservation of antimicrobial activity, and mechanical properties comparable to those of unmodified formulations [35,36,37]. However, they generally require the synthesis of photopolymerizable CS derivatives (e.g., via GMA modification) [30,31] and/or the incorporation of additional comonomers such as PEGDA [38,39] as formulation steps.

In contrast, phosphorylation has emerged as an alternative strategy for tailoring CS properties while preserving the original composition of commercially available resin systems. The introduction of phosphate groups (–PO_4_^3−^) modifies the physicochemical characteristics of CS while maintaining its overall cationic nature [20,21], thereby potentially preserving its contact-dependent antibacterial behavior. Unlike methacrylation approaches that primarily focus on improving polymer compatibility, phosphorylation enables the incorporation of a CS derivative while preserving antibacterial potential without requiring substantial modifications to the resin formulation. Supporting this concept, previous studies demonstrated that incorporating phosphorylated chitosan (P-Chi) into experimental light-cured composite resin systems promoted bioactivity and dentin remineralization while maintaining acceptable physicochemical and mechanical properties [40]. Nevertheless, evidence regarding the incorporation of P-Chi into vat-photopolymerized dental resins remains scarce. Therefore, this study aimed to investigate whether the incorporation of P-Chi into ST and LT vat-photopolymerized provisional resins could reduce bacterial activity while maintaining physicochemical and mechanical properties within clinically acceptable ranges.

## 2. Materials and Methods

### 2.1. Experimental Design

This in vitro study was designed to develop and characterize 3D-printed ST and LT printable resins modified with P-Chi at different concentrations. Six groups were defined: G1 = ST unmodified resin (control); G2 = LT unmodified resin (control); G3 = ST resin with 0.25% (*w*/*w*) P-Chi; G4 = LT resin with 0.25% (*w*/*w*) P-Chi; G5 = ST resin with 0.5% (*w*/*w*) P-Chi; and G6 = LT resin with 0.5% (*w*/*w*) P-Chi.

Specimens were fabricated using a standardized 3D printing protocol in accordance with the manufacturer’s recommendations. The experimental unit was defined according to the specific requirements of each test. Disk-shaped specimens (Ø12 mm × 3 mm) were used for surface, physicochemical, and optical analyses, whereas rectangular specimens (25 × 2 × 2 mm) were prepared for flexural strength testing following the International Organization for Standardization (ISO) 10477:2020 [41,42].

The primary outcome was the development of P-Chi-modified resins and their physicochemical, surface, mechanical, and optical characterization, while the secondary outcome comprised antibacterial performance against *S. mutans*.

#### 2.1.1. Specimen Sampling

Sample size allocation and analytical procedures were determined based on previous investigations employing comparable materials and outcome measures [14,41,43,44]. Variations in specimen number were attributed to the specific requirements and established practices of each analytical method. Thus, specimens per group were distributed as follows: SEM/FTIR/CLSM (*n* = 1; qualitative/descriptive characterization) [43]; profilometric roughness (Ra) and Knoop microhardness (KHN) (*n* = 30) [45]; contact angle (θ), color change (ΔE* and ΔE00), and flexural strength (*n* = 11) [41]; agar diffusion assay (*n* = 3) [44]; and colony-forming unit (CFU) counting (*n* = 9) [14,45].

#### 2.1.2. Blinding and Randomization

Specimens were randomly allocated to experimental groups using an electronic randomization tool (Random.org, Dublin, Ireland). Outcome assessors were blinded to group allocation. The operator responsible for mechanical and surface measurements was unaware of group allocation, and instrumental analyses were conducted by an independent researcher.

### 2.2. Synthesis and Characterization of P-Chi Nanoparticles

**Figure 1:**
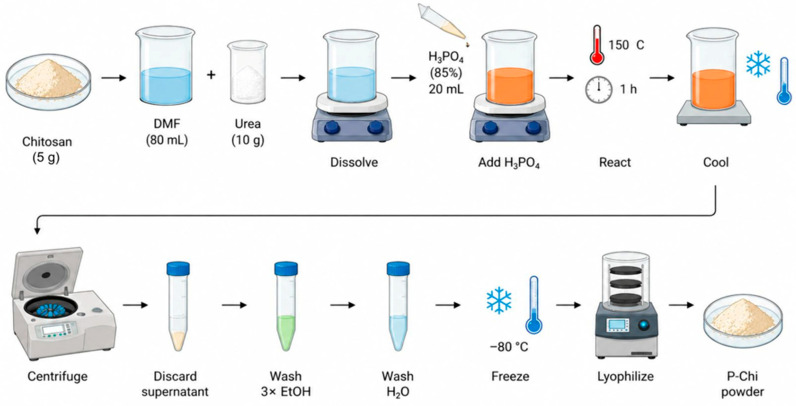
Workflow of P-Chi synthesis. CS was reacted with phosphoric acid in the presence of urea and DMF at 150 °C for 1 h, followed by centrifugation, ethanol and water washing, freezing, and lyophilization to obtain P-Chi powder.

The phosphorylation of CS was carried out as previously described by Ururahy et al., 2025 [46], who used P-Chi in dental research (Figure 1). P-Chi was synthesized by direct phosphorylation of low-molecular-weight CS (Sigma-Aldrich, St. Louis, MO, USA; 75–85% degree of deacetylation). Briefly, 5.00 g of CS was dispersed in 80 mL of anhydrous dimethylformamide (DMF; Êxodo Científica, Sumaré, Brazil) under magnetic stirring at room temperature until a homogeneous suspension was obtained. Subsequently, 10.00 g of urea (Synth, Diadema, Brazil) was then added and dissolved under continued stirring. Afterwards, 20 mL of phosphoric acid (85%, *w*/*w*) (Êxodo Científica, Sumaré, Brazil) was added dropwise to the suspension under vigorous stirring. The reaction vessel was loosely capped to minimize solvent evaporation while maintaining open atmospheric conditions, heated to 150 °C with continuous magnetic stirring, and held at this temperature for 1 h to allow the phosphorylation reaction to proceed.

After completion of the reaction, the mixture was allowed to cool to room temperature. Due to the high viscosity of the reaction mixture, the product was recovered by centrifugation (4000 rpm, 20 min, room temperature. The supernatant was discarded, and the precipitate was washed sequentially with anhydrous ethanol (3 × 50 mL) with centrifugation between each wash to recover the pellet, followed by washing with distilled water until the supernatant reached approximately neutral pH. The pellet was frozen at −80 °C and lyophilized. Based on the CS repeating unit, the molar ratio of CS:urea:phosphoric acid was approximately 1:5.4:9.4.

**Figure 2:**
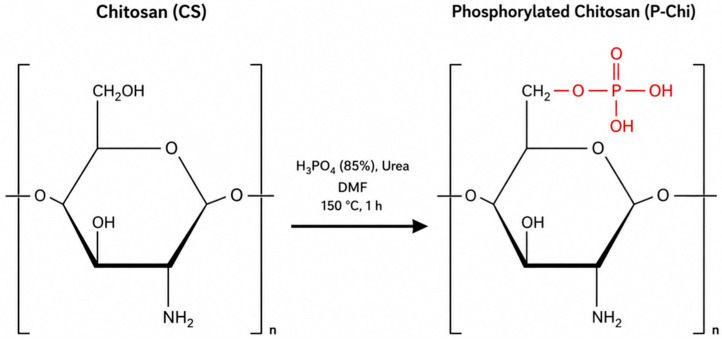
Proposed reaction scheme for the synthesis of P-Chi from CS using phosphoric acid, urea, and dimethylformamide (DMF) at 150 °C for 1 h.

The final product (3.80 g) was stored in a desiccator at room temperature under dry conditions until use. The average yield of the phosphorylation reaction in weight was 76%. The overall reaction is expressed in Figure 2.

#### Particle Size and Zeta Potential

Hydrodynamic diameter and ζ-potential were determined by dynamic light scattering (DLS) using a Zetasizer Nano ZS (λ = 580 nm, scattering angle 172°; Malvern Instruments, UK). Samples were diluted (1 mg/mL) in acetic acid prior to the analysis.

### 2.3. Incorporation of P-Chi Nanoparticles into 3D-Printed Resin

Phosphorylated CS nanoparticles were incorporated into the ST (G3, G5) and LT (G4, G6) resins. The ST resin (PriZma 3D Bio Prov, Makertech Labs, Tatuí, Brazil) is based on a mixture of methacrylic acid esters, comprising acrylic oligomers (>50% *w*/*w*), acrylic monomers (20–40% *w*/*w*), and proprietary photoinitiators and additives (≤2% *w*/*w*). The LT resin (PriZma 3D Bio Crown, Makertech Labs Tatuí, Brazil) is a methacrylate-based composite resin containing methacrylated monomers (>10% *w*/*w*), urethane dimethacrylate (UDMA; CAS 72869-86-4; <75% *w*/*w*), amorphous silica (≤8% *w*/*w*), ceramic fillers (<15% *w*/*w*), silanized zirconia (<2% *w*/*w*), titanium dioxide (<0.5% *w*/*w*), and TPO as a photoinitiator (<5% *w*/*w*). A key distinction between the two formulations lies in their complexity and filler content: the LT resin incorporates a more elaborate inorganic filler system, including UDMA, zirconia, titanium dioxide, and ceramic fillers. The exact chemical identities and concentrations of proprietary components were not disclosed by the manufacturer.

P-Chi was incorporated at concentrations of 0.25% and 0.5% by weight (%). These concentrations were selected based on the literature, which indicates that low CS concentrations can enhance antibacterial and mechanical properties [28]. To ensure proper dispersion of P-Chi within the 3D-printing resin, the nanoparticles were gradually incorporated into the liquid photopolymer resin under continuous magnetic stirring for 30 min until a visually homogeneous suspension was obtained [47,48,49].

### 2.4. Specimen Preparation

**Figure 3:**
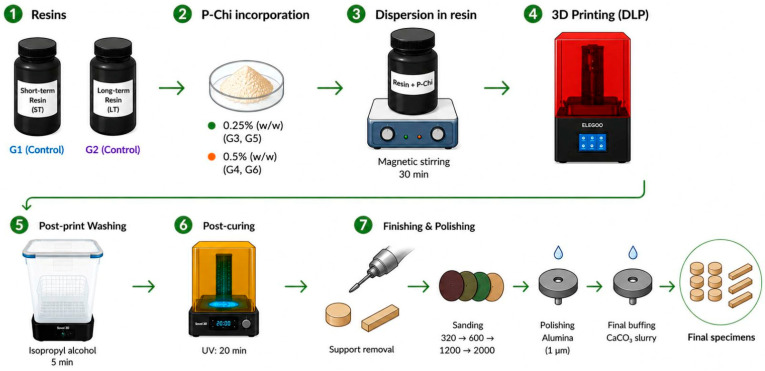
Schematic representation of resin modification and specimen fabrication. P-Chi was incorporated into ST and LT photopolymer resins at concentrations of 0.25 wt% and 0.50 wt%, followed by magnetic stirring for 30 min. Specimens were manufactured by digital light processing (DLP), washed in isopropyl alcohol, post-cured under UV light for 20 min, and subsequently finished and polished to obtain the final cylindrical and bar-shaped specimens used for physicochemical, mechanical, optical, and antibacterial analyses.

For the manufacturing of the 3D-printed resin specimens, digital designs were prepared using Rhinoceros software, version 6.0 (Robert McNeel & Associates, Seattle, WA, USA). The files were exported in STL (Standard Triangle Language) format and imported into Chitubox Basic 1.9.1 software (CBD Technology Co., Shenzhen, China), where support structures were added and the models were sliced into layers for printing. Samples were printed at a 90-degree orientation with a layer thickness of 0.05 mm. The exposure time was 15 s for the base layers at 80% light intensity and 5 s (±0.5 s) for subsequent layers. The printing parameters were established according to the manufacturer’s recommendations and adjusted after preliminary printing tests to ensure the dimensional accuracy of the final specimens. The specimens were printed using a Mars 4 printer (Elegoo, Shenzhen, China). After printing, specimens were washed in isopropyl alcohol for 5 min. Post-curing was performed for 20 min in a UV chamber (60 W, 167.71 mW/cm^2^, CuringOven, Done 3D, Ribeirao Preto, Brazil), according to the manufacturer’s instructions. After printing and post-curing, support structures were trimmed using a fine-cut bur coupled to a micromotor. All specimens underwent standardized mechanical sanding and polishing in a horizontal polishing machine (Panambra Industrial e Técnica SA, São Paulo, Brazil). Surface sanding was performed with sequential silicon carbide abrasive papers (320, 600, 1200, and 2000# grit; Norton Abrasivos Brasil, Guarulhos, Brazil), followed by polishing with a 1 μm alumina suspension (Fortel Indústria e Comércio Ltd.a., São Paulo, Brazil) and final buffing with a calcium carbonate aqueous slurry (Quimidrol Comércio, Indústria e Importação Ltd.a., Joinville, Brazil) [50]. All processes are schematically described in Figure 3.

### 2.5. Surface Characterization

#### 2.5.1. Scanning Electron Microscopy

Surface morphology and elemental composition of the samples were analyzed by scanning electron microscopy (SEM). Analyses were performed using a JSM-6610LV microscope (JEOL Ltd., Tokyo, Japan) operating in backscattered electron (BSE) mode at magnifications of 750×, 5000×, 17,500×, and 32,500×.

#### 2.5.2. Structural Characterization by Fourier-Transform Infrared Spectroscopy

Structural characterization of the 3D-printed resins was performed by FTIR to qualitatively compare spectra before and after CS incorporation. Analyses were carried out using an RFS 100/S spectrophotometer (Bruker Optics Inc., Billerica, MA, USA) in transmission mode. Spectra were acquired in the 4000–1000 cm^−1^ range at a resolution of 4 cm^−1^ under a nitrogen atmosphere. The evaluation focused on characteristic methacrylate bands (C=O, C–O–C, C–H) and CS-related functional groups (–OH, –NH_2_). No quantitative degree of conversion was assessed.

#### 2.5.3. Confocal Laser Scanning Microscopy

Confocal laser scanning microscopy (CLSM) (LEXT OLS4100^®^, Olympus Corp., Tokyo, Japan) was used to enable three-dimensional surface characterization. Measurements were performed at the center of each specimen using ×10 and ×100 objectives (216× total magnification) and a scanning area of 1280 × 1279 µm [45,50]. For each specimen, three images were acquired from distinct surface regions. Data processing was conducted using the instrument’s native OLS4100^®^ software (version 3.2; Olympus Corp., Tokyo, Japan), which also generated two- and three-dimensional topographic maps for qualitative analysis.

#### 2.5.4. Wettability

The wettability of 3D-printed resins was assessed by measuring the contact angle (θ) at room temperature. Measurements were obtained from three representative regions of the same specimen using an optical goniometer (OCA 20, DataPhysics Instruments GmbH, Filderstadt, Germany) with the sessile drop method using distilled water (3 μL) as the test liquid. The value of θ was calculated as the average of the measurements obtained from the equipment software using the Young–Laplace equation [50].

### 2.6. Mechanical Tests

#### 2.6.1. Surface Roughness

Surface roughness was evaluated using a profilometer (Surftest SJ-201P; Mitutoyo Corp., Kawasaki, Japan). Three measurements were obtained from the polished surface of each specimen, and the arithmetic mean roughness (Ra, μm) was calculated. Analyses were performed using a cutoff length of 0.8 mm, an evaluation length of 4.0 mm, and a scanning speed of 0.5 mm/s [51].

#### 2.6.2. Knoop Microhardness

Knoop microhardness was measured using a microhardness tester (HMV-2, Shimadzu, Kyoto, Japan) under a 50 gf load for 15 s. Three standardized indentations were performed linearly at equidistant intervals in the central and terminal regions of each specimen, and the mean value was recorded.

#### 2.6.3. Flexural Strength

Three-point flexural strength and elastic modulus were evaluated using bar-shaped specimens (25 × 2 × 2 mm), fabricated according to ISO 10477:2020 [42]. Mechanical testing was performed in a universal testing machine (EMIC MEM 2000, EMIC Equipamentos e Sistemas de Ensaio Ltd.a., São José dos Pinhais, Brazil) at a crosshead speed of 1 mm/min until fracture. Flexural strength (FS, MPa) was calculated as follows: FS = 3FL/2bh2, where F is the fracture load (N), L the support span (20 mm), b the specimen width, and h the specimen thickness (mm). The elastic modulus (GPa) was obtained from the slope of the linear elastic region. Mean values were statistically analyzed.

### 2.7. Objective Color Evaluation—CIE L*a*b* and CCIEDE 2000 System

Objective color assessment was performed using a calibrated portable spectrophotometer (SP62S, X-Rite Incorporated, Neu-Isenburg, Germany) coupled with QA-Master I software (version 5.5, X-Rite Incorporated, Neu-Isenburg, Germany), following established protocols [52,53]. Measurements were conducted under standard D65 illumination in a controlled environment (23 ± 1 °C). The probe was positioned perpendicular to the center of each polished specimen, and readings were obtained from each sample.

To evaluate color changes in the CIE L*a*b* system, the parameters L*, a*, and b* were recorded. The overall color difference (ΔE*) between groups was calculated using the following Equation (1):

(1) $$\Delta E^{*}=\sqrt{(\Delta L^{*}{)}^{2}+(\Delta a^{*}{)}^{2}+(\Delta b^{*}{)}^{2}}$$

For the CIEDE2000 metric, color differences were calculated using the ΔE_00_ Formula (2), with the parametric factors set to k_L = k_C = k_H = 1, as shown below.

(2) $$\Delta E_{00}=\sqrt{{\left(\frac{\Delta L^{\prime }}{k_{L}S_{L}}\right)}^{2}+{\left(\frac{\Delta C^{\prime }}{k_{C}S_{C}}\right)}^{2}+{\left(\frac{\Delta H^{\prime }}{k_{H}S_{H}}\right)}^{2}+R_{T}(\frac{\Delta C^{\prime }}{k_{C}S_{C}})(\frac{\Delta H^{\prime }}{k_{H}S_{H}})}$$

### 2.8. Antibacterial Performance—Agar Diffusion Test

Antibacterial activity was assessed using an agar diffusion assay (*n* = 3) with *S. mutans* (ATCC 25175), following previously reported protocols [44]. Brain Heart Infusion (BHI) agar (Kasvi, São José dos Pinhais, Brazil) was prepared, sterilized, and dispensed into Petri dishes (25 mL per plate).

Bacterial cultures were grown in Tryptic Soy Broth (TSB, 10 mL) under anaerobic conditions for 24 h. The suspensions were centrifuged (4200× *g*, 5 min), washed three times with phosphate-buffered saline (PBS), and resuspended in PBS. Cell density was adjusted to 10^8^ CFU/mL (OD = 0.150 at 625 nm), and 500 µL of the inoculum was uniformly spread onto the agar surface.

Plates were divided into quadrants. In one plate, specimens from groups G1, G3, G5, and a chlorhexidine digluconate 0.12% control were positioned, while a second plate contained groups G2, G4, G6, and the same control. All specimens were placed in direct contact with the agar surface. Plates were incubated at 37 °C for 48 h under anaerobic conditions. After incubation, inhibition zones were documented, and their diameters measured using a digital caliper.

### 2.9. Antibacterial Performance—Colony-Forming Unit (CFU) Count

The antibacterial performance of the functionalized surfaces (*n* = 9) was evaluated by quantifying *S. mutans* (ATCC 25175) biofilms using the colony-forming unit (CFU) method, as previously described [14,45]. All incubations were performed under microaerophilic conditions.

Specimens were washed with water and detergent, then immersed in alcohol on both sides. After air-drying, the specimens were exposed to ultraviolet (UV) light for 30 min per side, and the sterilization efficacy was confirmed by incubation in Brain Heart Infusion (BHI) broth (Kasvi, São José dos Pinhais, Brazil) at 37 °C for 14 days.

*S. mutans* was reactivated on BHI agar (Kasvi, São José dos Pinhais, Brazil) for 48 h, transferred to BHI broth, and incubated for 24 h. The culture was centrifuged (4200× *g*, 5 min), washed with phosphate-buffered saline (PBS), and adjusted to 10^8^ CFU/mL (OD = 0.150 at 625 nm).

Under aseptic conditions, specimens were placed in 24-well plates containing 1.5 mL of inoculated medium, except for the negative control (sterile medium). Plates were incubated at 37 °C under microaerophilic conditions with agitation (75 rpm) for 90 min to allow adhesion. After washing with PBS, fresh medium was added, and biofilms were developed for 24 h with partial medium renewal after 24 h.

Biofilms were detached by ultrasonication (40 kHz, 200 W, 20 min), followed by vortexing. Serial dilutions (10^0^ to 10^−8^) were plated on Mitis Salivarius agar (BD Difco, Becton, Dickinson and Company, Sparks, MD, USA) supplemented with 20% sucrose and 50 IU of bacitracin (Sigma-Aldrich, St. Louis, MO, USA). CFUs were counted and expressed as log_10_ CFU/mL, considering plates with 1–300 colonies.

### 2.10. Statistical Analysis

Statistical analysis was performed at a 5% significance level (α = 0.05). Data normality and homogeneity of variances were assessed using the Shapiro–Wilk and Levene’s tests, respectively. Parametric data were analyzed by one-way ANOVA followed by Duncan’s post hoc test (surface roughness, microhardness, and flexural strength), while independent samples were compared using Student’s *t* test (ΔE_00_). All analyses were conducted using SPSS for Windows (version 20.0; IBM Corp., Armonk, NY, USA).

## 3. Results

### 3.1. Nanoparticles Characterization

**Figure 4:**
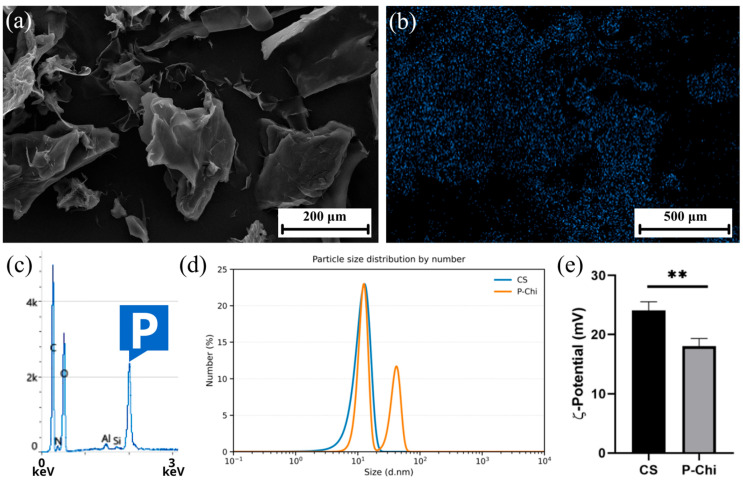
Morphological, compositional, and physicochemical characterization of CS and P-Chi. (**a**) SEM micrograph of P-Chi particles. (**b**) Phosphorus elemental mapping of P-Chi. (**c**) Representative EDS spectrum confirming phosphorus incorporation after phosphorylation. (**d**) Number-based particle size distribution determined by DLS. (**e**) ζ-potential of CS and P-Chi. Error bars represent the standard deviation. ** *p* < 0.01.

Figure 4 summarizes the morphological, compositional, and physicochemical characterization of CS before and after phosphorylation. SEM analysis revealed irregular lamellar structures with folded surfaces, characteristic of dried CS particles (Figure 4a). Elemental mapping showed a homogeneous distribution of phosphorus in the modified material (Figure 4b), while EDS confirmed the presence of phosphorus-related signals in P-Chi, supporting the successful incorporation of phosphate groups (Figure 4c).

Dynamic light scattering (DLS) analysis (Figure 4d) revealed similar predominant nanoparticle populations for CS and P-Chi, centered at 12.77 and 12.49 nm, respectively. However, phosphorylation altered the particle size distribution, resulting in the appearance of an additional population centered at 41.78 nm in the P-Chi suspension, suggesting changes in colloidal organization.

Zeta potential analysis (Figure 4e) demonstrated a significant reduction in surface charge after phosphorylation (*p* < 0.01). The ζ-potential decreased from approximately +24 mV for CS to +18 mV for P-Chi while maintaining an overall positive surface charge. This reduction is consistent with the introduction of phosphate groups, which partially neutralized the positively charged amino groups of CS. Collectively, the SEM, EDS, DLS, and ζ-potential findings provide complementary evidence of successful CS phosphorylation, indicating that P-Chi preserved its cationic character while exhibiting altered colloidal organization that may influence its interactions with the resin matrix.

### 3.2. Scanning Electron Microscopy (SEM)

**Figure 5:**
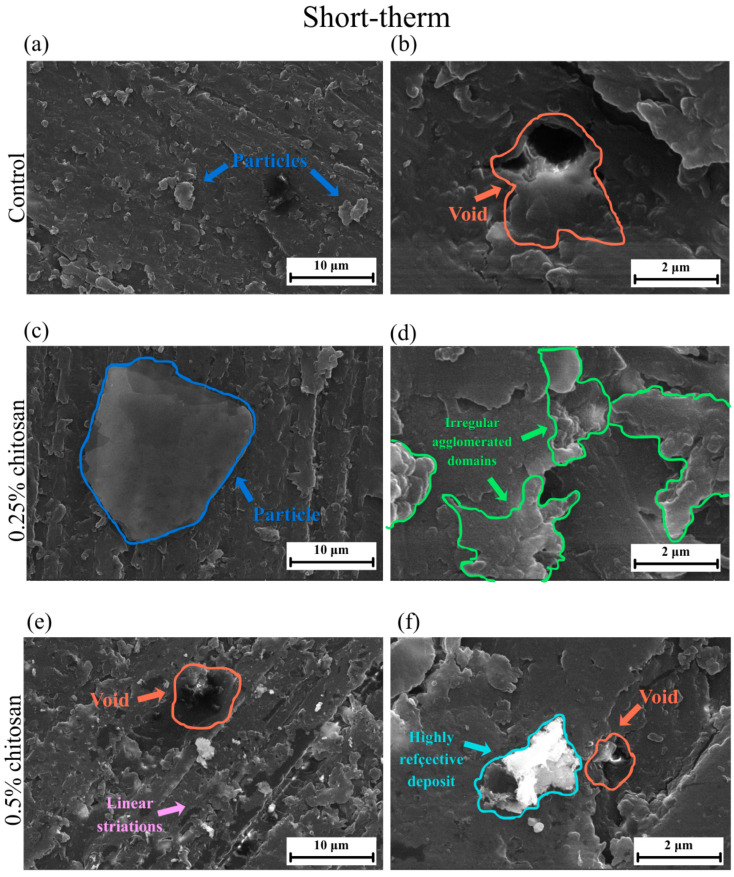
SEM images of ST resins. Representative scanning electron microscope images of control and nanocomposite resins surface (magnification ×5000 and ×32,500). (**a**,**b**) ST unmodified resin (control); (**c**,**d**) ST resin with 0.25% (*w*/*w*) P-Chi; and (**e**,**f**) ST resin with 0.5% (*w*/*w*) P-Chi.

**Figure 6:**
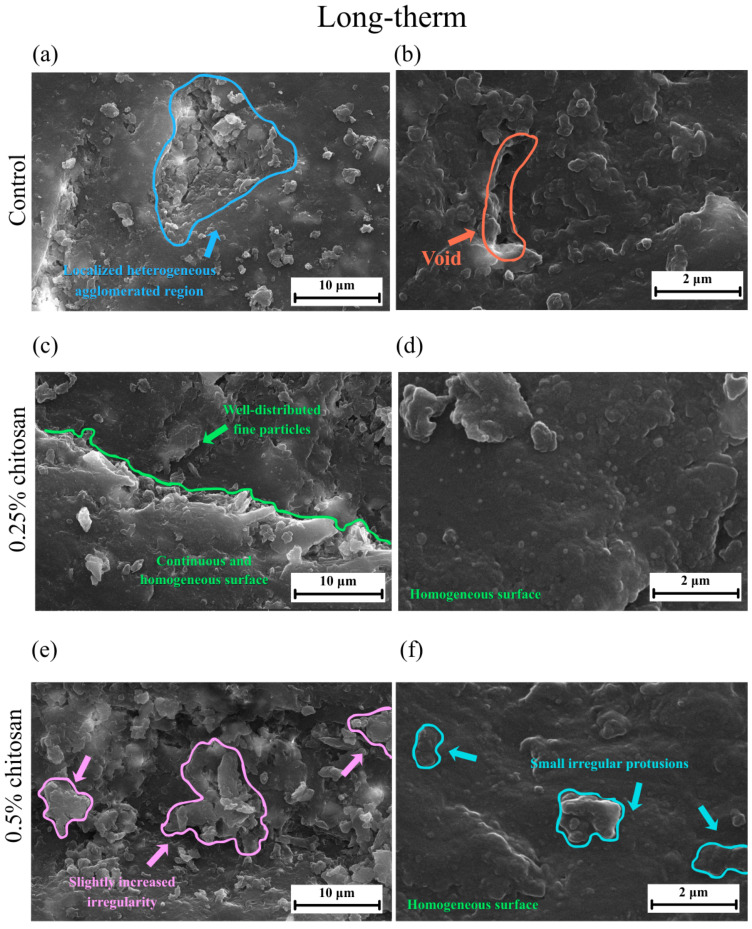
SEM images of LT resins. Representative scanning electron microscope images of control and nanocomposite resins surface (magnification ×5000 and ×32,500). (**a**,**b**) LT unmodified resin (control); (**c**,**d**) LT resin with 0.25% (*w*/*w*) P-Chi; and (**e**,**f**) LT resin with 0.5% (*w*/*w*) P-Chi.

Representative SEM images of the specimens of ST and LT resin are presented in Figure 5 and Figure 6, respectively. Overall, SEM analysis revealed distinct differences in surface morphology between resin types and P-Chi concentrations.

The ST control group showed a relatively flat surface, although scattered particles (Figure 5a), superficial irregularities, and a large void suggestive of a polymerization defect were observed (Figure 5b). The 0.25% P-Chi group exhibited a heterogeneous surface with a prominent faceted particle (Figure 5c), indicating possible P-Chi agglomeration and incomplete dispersion (Figure 5d). At 0.50%, the surface became more irregular, with linear striations (Figure 5e), voids, and a large, highly reflective deposit consistent with phase-separated P-Chi domains, suggesting reduced compatibility with the ST resin matrix at the higher concentration (Figure 5f). The highly reflective regions observed under BSE mode may also suggest localized compositional heterogeneity associated with P-Chi-rich domains.

The LT control group exhibited scattered particles and a localized heterogeneous agglomerated region (Figure 6a), while a superficial void-like defect was observed at higher magnification (Figure 6b). The LT resin appeared to accommodate the incorporation of P-Chi more uniformly, particularly at 0.25% (Figure 6c,d), where the surface exhibited the greatest degree of homogeneity and continuity among all groups evaluated, with well-distributed fine particles and smoother surface regions. At 0.50% (Figure 6e,f), the surface remained relatively continuous but showed a slight increase in irregularity and the presence of small irregular protrusions compared with the 0.25% group, indicating reasonably good distribution with some reduction in surface regularity.

These findings suggest that the compatibility between P-Chi and the resin matrix is influenced by both the type of resin and the concentration of the additive, with the LT formulation demonstrating superior incorporation at the concentrations tested.

### 3.3. Structural Characterization by Fourier-Transform Infrared Spectroscopy (FTIR)

**Figure 7:**
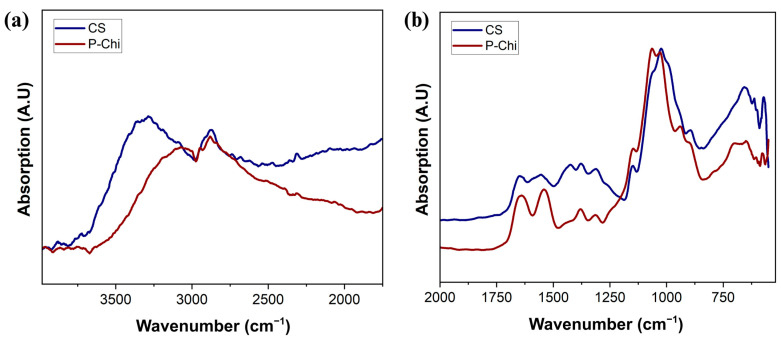
Confirmatory spectra of P-Chi nanoparticles in the regions from 4000 to 1750 cm^−1^ (**a**) and from 2000 to 550 cm^−1^ (**b**).

Figure 7a,b show the FTIR spectra in the 4000–1750 and 2000–550 cm^−1^ regions, respectively. The comparison between spectra confirms the phosphorylation of CS. Both CS and P-Chi showed characteristic CS bands, including broad O–H/N–H stretching vibrations around 3200–3500 cm^−1^ and C–H stretching near 2920 cm^−1^ in Figure 7a. The spectrum of the CS sample showed typical bands, such as Amide I (~1650) and Amide II (~1590).

Regarding the phosphorylation (sample P-Chi), a reduction in the 3200 band is observed (Figure 7a). Moreover, other small changes are observed, such as an increase in the absorption intensity in the 1200–900 cm^−1^ region, particularly around 1050–1100 cm^−1^, corresponding to P=O and P–O–C stretching vibrations (Figure 7b). These changes indicate the incorporation of phosphate groups into the CS structure [54]. The appearance or intensification of a band around ~1640 and 1650 in phosphorylated derivatives may be related to the deformation vibration of the protonated amino groups or to interactions between phosphate groups and the amines.

**Figure 8:**
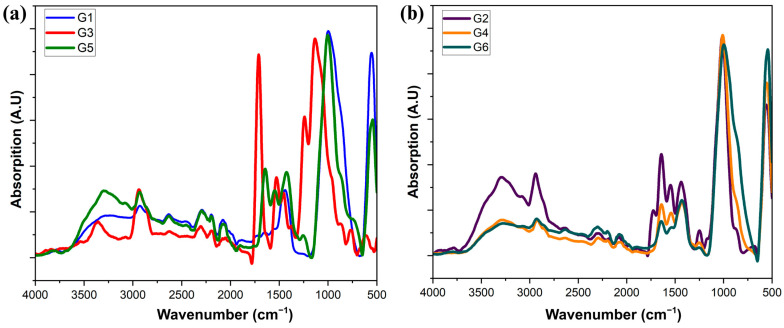
Comparative spectra of the control specimens and the groups incorporating 0.25% and 0.5% P-Chi nanoparticles in the region from 4000 to 500 cm^−1^ for ST (**a**) and LT resins (**b**).

Figure 8 shows the FTIR spectra of all groups. FTIR analysis confirmed the successful incorporation of P-Chi into both the ST and LT resin matrices, as evidenced by the emergence or intensification of absorption bands associated with P=O stretching (~1250 cm^−1^) and P–O–C stretching (~1050 cm^−1^) in all modified groups (samples G3, G5, G4 and G6).

The nature and extent of the spectral modifications, however, differed between resin types. In the ST resin (Figure 8a), concentration-dependent differences were apparent, most notably the exclusive presence of a band at ~2100 cm^−1^ in G3 and the distinct spectral profiles of G3 and G5, suggesting that the mode of P-Chi incorporation is sensitive to concentration in this matrix. In the LT resin (Figure 8b), G4 and G6 exhibited highly similar spectral profiles, suggesting a concentration-independent chemical interaction with the matrix, consistent with the suppression of O–H stretching observed in this resin type.

### 3.4. Confocal Laser Scanning Microscopy (CLSM)

**Figure 9:**
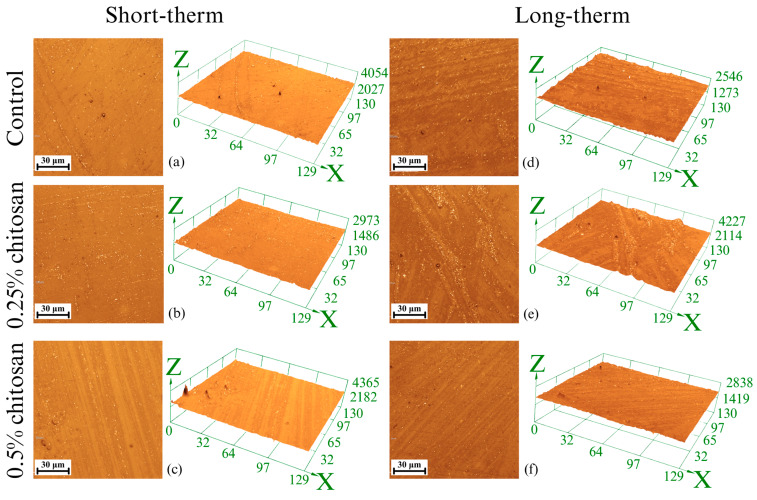
Representative CLSM 2D images (10×) and corresponding 3D surface reconstructions (100×) of short-term (ST) and long-term (LT) 3D-printed resins: (**a**) ST control; (**b**) ST + 0.25% P-Chi; (**c**) ST + 0.50% P-Chi; (**d**) LT control; (**e**) LT + 0.25% P-Chi; and (**f**) LT + 0.50% P-Chi.

Representative 2D and 3D CLSM images are shown in Figure 9. All groups exhibited relatively homogeneous surfaces with parallel polishing grooves and isolated irregularities.

The ST control group (Figure 9a) exhibited a relatively flat, homogeneous surface topography. The 2D map revealed only isolated point-like depressions, while the 3D reconstruction confirmed a largely planar relief with no prominent elevations or directional patterning. Incorporation of 0.25% P-Chi (Figure 9b) resulted in a surface morphology broadly comparable to that of the control group. The 2D map showed a uniform distribution of small bright surface features, potentially associated with dispersed P-Chi particles or surface microfeatures, without evidence of large-scale agglomeration. At the higher concentration of 0.50% (Figure 9c), the surface exhibited more pronounced directional linear features and localized topographic irregularities. Parallel linear striations were also evident in the 2D map, likely attributable to mechanical deformation or specimen preparation artifacts. These findings suggest that, at this concentration, localized accumulation or incomplete dispersion of the additive may occur within the ST matrix.

The LT control group (Figure 9d) exhibited a relatively homogeneous surface; however, compared with the ST control (Figure 9a), more evident directional striations were observed, suggesting subtle surface heterogeneity. Incorporation of 0.25% P-Chi (Figure 9e) resulted in a more irregular morphology than that observed for the corresponding ST group (Figure 9b), characterized by a greater density of bright surface features and gentle localized elevations. These findings suggest a distinct interaction between P-Chi and the LT resin matrix, potentially related to differences in polymer network organization or additive dispersion during curing. Conversely, specimens containing 0.50% P-Chi (Figure 9f) exhibited the smoothest surface appearance among the LT groups, with no evident major morphological alterations. Nevertheless, subtle unidirectional striations remained evident, like those observed in Figure 9c, likely due to specimen preparation artifacts.

### 3.5. Surface Roughness

Surface roughness results are presented in Table 1 and showed significant differences among the experimental groups (Kruskal–Wallis, H = 141.2, *p* < 0.001). The lowest Ra values were observed for G1 (0.036 ± 0.019 µm) and G2 (0.031 ± 0.007 µm), with no statistically significant difference between these groups (*p* > 0.05).

In contrast, higher roughness values were found for G3 (0.192 ± 0.106 µm), G4 (0.364 ± 0.145 µm), G5 (0.332 ± 0.154 µm), and G6 (0.232 ± 0.123 µm). G4 exhibited the highest Ra values, although no significant difference was observed compared with G5 (*p* > 0.05). However, G4 showed significantly greater roughness than G6 (*p* = 0.004). No statistically significant differences were identified among G3, G5, and G6 (*p* > 0.05).

### 3.6. Knoop Microhardness

Knoop microhardness was evaluated on disk-shaped specimens to investigate the effect of incorporating CS at concentrations of 0.25 and 0.5% (*w*/*w*) and the results are presented in Table 2. Microhardness values showed a normal distribution and homogeneity of variances (*p* > 0.05). One-way ANOVA revealed a statistically significant difference among the groups (F(5,174) = 30.58, *p* < 0.0001).

G4 presented the lowest microhardness (14.48 ± 2.32 KHN), significantly differing from all other groups (*p* < 0.0001). The remaining groups (G1: 19.36 ± 2.06, G2: 20.12 ± 0.94, G3: 19.14 ± 2.48, G5: 19.46 ± 1.49, and G6: 20.27 ± 0.82 KHN) showed no significant differences among themselves (*p* > 0.05), except for a slight but significant difference between G3 and G6 (*p* = 0.042), with G6 presenting higher values.

### 3.7. Wettability

**Figure 10:**
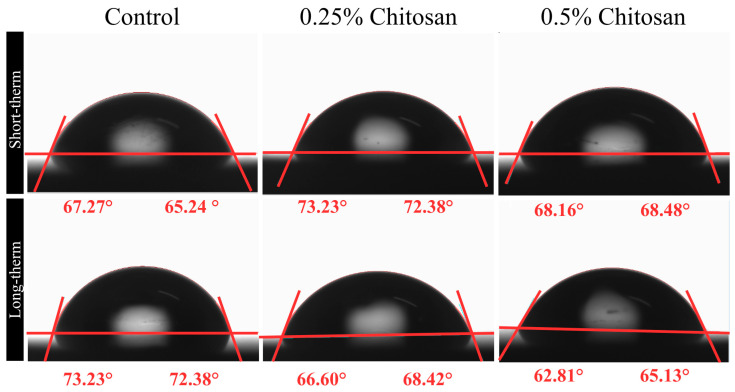
WCA (θ, °) of the surfaces of ST and LT resins at different concentrations (0, 0.25, and 0.5%) in distilled water.

The WCA (θ, °) results are presented in Table 3 and Figure 10. Significant differences among the experimental groups were observed (Welch’s ANOVA, *p* < 0.001). G1 exhibited the highest contact angle value (73.07 ± 10.55°), whereas G6 showed the lowest value (61.18 ± 2.02°). All groups presented contact angle values below 90°, indicating hydrophilic behavior. Significant differences were identified between G6 and G1, G4, and G5 (*p* < 0.05). No statistically significant differences were observed among G2, G3, G4, and G5 (*p* > 0.05).

### 3.8. Flexural Strength

The mechanical properties obtained from the three-point bending test are summarized in Table 4. Flexural strength was significantly influenced by both concentration and resin type. (Welch’s ANOVA, *p* < 0.001). G1 and G2 exhibited the highest flexural strength values (118.13 ± 8.54 MPa and 119.13 ± 8.29 MPa, respectively), with no statistically significant difference between them (*p* > 0.05). In contrast, G3 and G4 showed the lowest values (95.55 ± 7.85 MPa and 96.83 ± 12.89 MPa, respectively), also without significant differences between these groups (*p* > 0.05). G5 and G6 demonstrated intermediate flexural strength values (108.13 ± 11.73 MPa and 112.43 ± 13.87 MPa, respectively).

### 3.9. Objective Color Evaluation—CIE L*a*b* and CIEDE2000 System

Color variation results for the CIE L*a*b* and CIEDE2000 systems are presented in Table 5. The evaluation of color variation showed that, for the CIE L*a*b* system, significant differences were detected among the groups (*p* < 0.001). Lower ΔE* values were observed for G3 (1.21 ± 0.67) and G5 (0.97 ± 0.16), which showed no statistically significant difference (*p* > 0.05). Higher color variation values were found for G4 (3.67 ± 1.02) and G6 (3.53 ± 0.26), both of which differed significantly from G3 and G5 (*p* < 0.05), while no significant difference was observed between G4 and G6 (*p* > 0.05).

Similarly, for the CIEDE2000 system, statistically significant differences were observed among the groups (*p* < 0.001). G3 (0.82 ± 0.52) and G5 (0.65 ± 0.10) exhibited the lowest color variation values and did not differ significantly from each other (*p* > 0.05). In contrast, G4 (2.96 ± 0.77) and G6 (2.94 ± 0.11) showed significantly higher ΔE_00_ values compared with G3 and G5 (*p* < 0.05), with no significant difference between G4 and G6 (*p* > 0.05). Overall, G4 and G6 showed the greatest color alteration across all color evaluation systems.

### 3.10. Antibacterial Performance—Agar Diffusion Test

**Figure 11:**
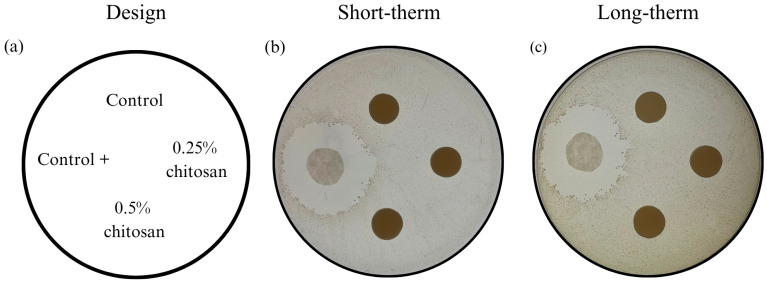
Petri dishes after the agar diffusion assay: (**a**) Distribution of the disks (Unmodified resin—negative control, 0.25%, and 0.5% P-Chi, and positive control—chlorhexidine); (**b**) ST resin (**c**) LT resin, showing the absence of inhibition halos around the tested surfaces and well-defined halos only around the positive control.

The agar diffusion test is demonstrated in Figure 11. They showed no inhibition halos in all groups, except in the positive control containing 0.12% chlorhexidine gluconate, indicating that P-Chi nanoparticles at concentrations of 0.25% and 0.5% did not exhibit antibacterial activity against *S. mutans* by diffusible substances.

### 3.11. Antibacterial Performance—Colony-Forming Unit (CFU) Count

**Figure 12:**
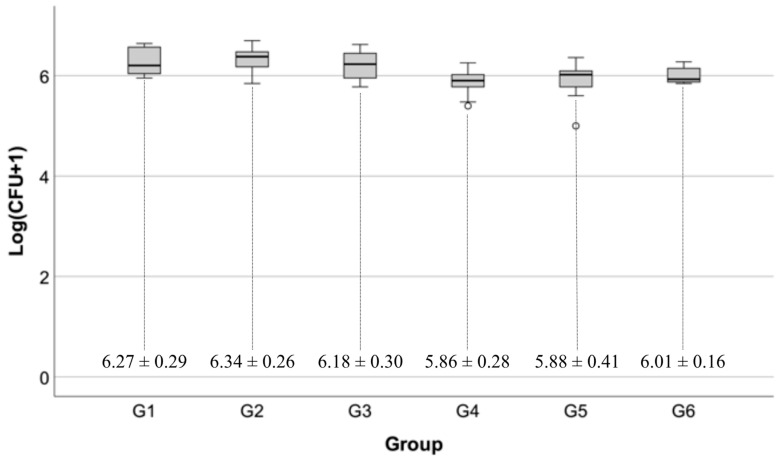
Boxplot representing Log(CFU + 1) values for the experimental groups (G1–G6). The central line within each box indicates the median, the box limits represent the interquartile range, and the whiskers correspond to the minimum and maximum non-outlier values. Circular markers indicate outliers. Numerical mean ± SD values are provided below each boxplot for descriptive purposes.

For the colony-forming unit (CFU) count analyses, data were initially assessed for normal distribution and homogeneity of variance. Since these assumptions were met, a two-way ANOVA was performed, followed by Bonferroni-adjusted multiple comparisons at the 5% significance level (Figure 12).

Resin type had no significant effect on Log(CFU + 1) values (F(1,45) = 0.094, *p* = 0.761, partial η^2^ = 0.002). In contrast, P-Chi concentration significantly affected bacterial counts (F(2,45) = 9.015, *p* < 0.001, partial η^2^ = 0.286), indicating a large effect size. Although the interaction between resin type and concentration did not reach statistical significance (F(2,45) = 3.046, *p* = 0.057), a small-to-moderate interaction effect was observed (partial η^2^ = 0.119) (Table 6).

Bonferroni-adjusted pairwise comparisons revealed that the 0% P-Chi group exhibited significantly higher Log(CFU + 1) values than both the 0.25% (*p* = 0.016) and 0.50% (*p* = 0.002) P-Chi groups. No statistically significant difference was observed between the 0.25% and 0.50% concentrations (*p* = 1.000). Cohen’s d values indicated large effect sizes for the comparisons between 0% and 0.25% P-Chi and between 0% and 0.50% P-Chi, whereas the difference between the modified groups was small (Table 7).

Although the absolute differences in log_CFU_ values were moderate, the modified groups demonstrated a consistent reduction in microbial load compared with the control group. Furthermore, no statistically significant differences were observed between the 0.25% and 0.50% concentrations, suggesting no dose-dependent effect within the evaluated concentration range.

## 4. Discussion

The present study evaluated the influence of P-Chi incorporation into ST and LT 3D-printed dental resins on surface, mechanical, optical, and antibacterial properties against *S. mutans*. The literature demonstrates that 3D-printed resins frequently exhibit lower flexural strength and surface hardness than milled or heat-polymerized materials [55,56,57,58], limiting their LT intraoral performance [11,59]. In this context, nanoparticles have been investigated as multifunctional additives capable of simultaneously modulating mechanical, physicochemical, and biological properties [41,47,60,61]. Nevertheless, the incorporation of P-Chi into 3D-printed photopolymerizable provisional resins remains poorly explored, despite its biofunctional potential to modify interfacial interactions, surface behavior, and antibacterial response.

To enhance the polymer–particle interfacial interaction between the printed resin matrices and the multifunctional nanoparticles, CS phosphorylation was employed. Cohen et al. (2022) [62] demonstrated that chemical modification of CS increases its solubility, cation-exchange capacity, and bioactivity compared with unmodified CS. In this context, phosphorylation was employed to modify the polymer–particle interface and potentially promote more stable interfacial interactions with the resin matrix. As described by Robin et al. (2026) [63], these structural modifications can be confirmed by FTIR spectroscopy. In the present study, increased absorption was observed within the 1200–900 cm^−1^ region, particularly between 1050 and 1100 cm^−1^, consistent with P=O and P–O–C vibrations, suggesting the incorporation of phosphate groups into the polymeric chain. These findings may indicate greater affinity of P-Chi for the organic matrix and alterations in surface energy, potentially favoring more stable matrix–particle interactions. Nevertheless, group G3 exhibited a specific band at around 2100 cm^−1^, possibly associated with residual chemical species or distinct molecular interactions within the composite, which may have contributed to interfacial heterogeneity and influenced the material’s mechanical and optical properties.

Thus, the efficiency of P-Chi incorporation and interaction may depend on the specific chemical composition of the resin matrix. In 3D printing resins, the formulation typically includes base monomers and oligomers, as well as diluents and photoinitiators, as described by Choi et al., 2022 [64]. According to Burgos-Mármol et al., 2017 [65], the specific monomers used in resin matrices influence nanoparticle behavior through their effects on polymer chain conformation and mobility. Consequently, polymer chain rigidity may affect the ability of nanoparticles to diffuse throughout the matrix. Liu et al., 2011 [66] also demonstrated that chemical compatibility between the resin matrix and nanoparticles is critical, since weak polymer–filler interactions may promote nanoparticle aggregation due to insufficient penetration into the matrix. Conversely, when these interactions are overly strong, aggregation may result from bridging effects between particles.

These factors may explain the formation of the specific band observed in the FTIR spectrum of group G3, which may be associated not only with the presence of P-Chi but also with the chemical nature of the ST resin itself. SEM analysis also revealed possible P-Chi agglomerates on the surface of G3, indicating incomplete nanoparticle dispersion. Similarly, group G5 exhibited a large surface deposit consistent with localized P-Chi accumulation, further suggesting lower compatibility with the ST resin matrix. CLSM analysis demonstrated relative preservation of topographic continuity in most groups, although localized surface elevations and linear defects were observed, particularly in G5. Momper et al. (2020) [67] showed that nanoparticle functionalization with surface-bound ligands chemically similar to the photoresin is crucial for achieving homogeneous dispersion. This strategy allows increased nanoparticle loading without inducing agglomeration during the 3D printing process. An example is the study by Liao et al., 2020 [68], which demonstrated that silanization of zirconium phosphate particles loaded with silver nanoparticles (6S-NP3) reduced the average size of particle agglomerates and significantly improved compatibility and distribution within denture base resin. In this context, phosphorylation may have contributed to improving CS dispersion within the resin matrix, given that unmodified CS is inherently insoluble in water and chemically incompatible with certain substances, as demonstrated by Biswas et al., 2025 [69]. However, P-Chi may still not have undergone sufficiently effective mechanical and physical dispersion. As reported by Shahbazi et al., 2024 [70], one of the main challenges in the use of nanomaterials is their natural tendency to form agglomerates or clusters due to strong hydrogen bonding and van der Waals interactions. Such aggregation may negatively affect the mechanical, thermal, and overall physicochemical properties of the final composite material.

Regarding surface roughness, P-Chi incorporation increased Ra values in all experimental groups, with G4 (0.364 ± 0.145), G5 (0.332 ± 0.154) and G6 (0.232 ± 0.123) exceeding the clinically acceptable thresholds reported in the literature (Ra < 0.2 μm) [71,72], while G3 remained close to the critical limit (0.192 ± 0.106). The increase in surface roughness observed after incorporating P-Chi may have important implications for bacterial adhesion and biofilm development [73,74]. Surfaces with an average roughness (Ra) greater than 0.2 μm are generally considered more susceptible to microbial colonization, as they provide a larger effective surface area and a greater number of contact points for bacterial attachment [73,75]. Furthermore, surface irregularities create protected microenvironments with reduced shear forces, protecting adhering bacteria from mechanical removal and facilitating biofilm maturation [76]. Increased roughness has also been associated with a reduction in the activation energy required for bacterial adhesion, making the attachment process energetically more favorable [75].

Interestingly, the findings of the present study did not follow the expected trend. Despite presenting surface roughness values above the threshold commonly associated with increased biofilm accumulation, the P-Chi-modified groups exhibited significantly lower *S. mutans* CFU counts than the unmodified controls (*p* = 0.001). Therefore, although increased roughness is traditionally regarded as a factor favoring bacterial adhesion, the present findings suggest that the chemical functionalization promoted by P-Chi may have exerted a more pronounced influence on microbial behavior than topography alone. In this context, the antibacterial activity associated with P-Chi appears to have outweighed the potential biofilm-promoting effect expected from the increase in surface roughness, indicating that surface chemistry may play a decisive role in modulating bacterial colonization.

Similar findings have been reported in chitosan-modified resins, in which surface behavior is directly influenced by the incorporation method, chemical functionalization, and interaction with the polymer matrix [27]. In the present study, the increased roughness may be attributed to incomplete nanoparticle dispersion and localized P-Chi agglomeration observed in the SEM analysis, although CLSM did not reveal pronounced large-scale topographic irregularities. This suggests that the elevated Ra values primarily reflect localized surface heterogeneities and possible nanoparticle exposure at the material–environment interface. Despite the two-step finishing and polishing protocol employed according to Son and Lee 2020 [77], most modified groups maintained Ra values above the clinically accepted threshold. However, Rojas-Rueda et al., 2025 [78] demonstrated that alternative post-processing strategies, such as diamond rubber polishing systems or the application of light-cured surface varnishes, can reduce surface roughness to below 0.2 μm, indicating that the increased roughness observed in the present study may be mitigated through optimized finishing procedures. Although linear profilometry (Ra) provides relevant information regarding surface heterogeneity and potential microbial retention, areal roughness analysis (Sa) could provide a more comprehensive topographical characterization. Nevertheless, superficial nanoparticle exposure may have contributed to the observed hydrophilic behavior and to modifications in protein–bacteria interactions during microbial adhesion.

Furthermore, the wettability values observed in the present study ranged from 61.18 to 73.07°, with all groups remaining within the hydrophilic range (<90°). Wuersching et al., 2026 [79], demonstrated that surface free energy and wettability play a more decisive role than surface roughness in bacterial adhesion. In this context, the improved antibacterial performance against *S. mutans* observed in the CFU assay likely resulted from the combined effects of changes in wettability, surface composition, and the exposure of functional groups associated with P-Chi.

Regarding mechanical properties, the flexural strength results showed that the incorporation of 0.25% P-Chi negatively affected mechanical resistance, whereas the groups containing 0.5% exhibited intermediate values closer to the control groups, particularly in the LT resin. Although all experimental groups exceeded the minimum flexural strength requirement of 80 MPa established by ISO 4049 [80], values approaching or exceeding 100–120 MPa are generally considered more desirable for restorations subjected to higher functional loads [81,82]. In contrast to the increases in flexural strength reported by ElMalah et al. (2024) [41] following nanofiller incorporation into 3D-printed resins, P-Chi incorporation in the present study resulted in reductions of 5.6–19.1%, particularly at 0.25 wt%, while the 0.50 wt% formulations approached the control values. The greatest reductions were observed at the 0.25 wt% concentration (−19.1% for ST and −18.7% for LT resins), whereas the 0.50 wt% formulations exhibited smaller changes (−8.5% and −5.6%, respectively), approaching the mechanical performance of the controls.

Despite these reductions, all modified groups remained above the ISO 4049 [80] threshold and within the range considered clinically acceptable for provisional restorative applications. These findings indicate that, unlike conventional reinforcing nanofillers, the mechanical effect of P-Chi is highly dependent on its interaction with the resin matrix and does not necessarily translate into enhanced flexural strength. Nevertheless, the P-Chi-modified formulations maintained mechanical performance comparable to that reported for commercially available materials. The lower microhardness and flexural strength observed in some P-Chi-containing groups may be associated with incomplete nanoparticle dispersion and localized interfacial heterogeneity, whereas the higher elastic modulus observed in G6 suggests increased structural rigidity without a proportional increase in ultimate flexural resistance. Interestingly, although G6 exhibited flexural strength values 5.6% lower than those of its corresponding control group (112.43 ± 13.87 MPa vs. 119.13 ± 8.29 MPa), it presented a 10.7% higher elastic modulus (3494.95 ± 301.30 MPa vs. 3156.18 ± 306.20 MPa). These findings suggest that the combined effect of the LT resin matrix and the higher P-Chi concentration may have increased structural rigidity without a proportional improvement in ultimate mechanical resistance, potentially reflecting alterations in stress redistribution dynamics within the composite.

In this context, the mechanical performance observed in the present study remains clinically acceptable and comparable to that reported for many commercially available 3D-printed resins. However, the literature consistently indicates that the mechanical behavior of additively manufactured resins is often at the lower limit of clinical expectations and remains inferior to that of CAD/CAM-milled materials, which exhibit superior mechanical, optical, and chemical stability [11,58].

When compared with nanoparticle-modified materials, recent evidence demonstrates that the mechanical and surface behavior of nanoparticle-modified PMMA systems critically depends on nanoparticle dispersion, chemical functionalization, and matrix–particle interfacial stability, factors that can simultaneously modulate structural heterogeneity, stress distribution, and the overall performance of the composite [27,83]. Additionally, studies involving 3D-printed resins have demonstrated that properties such as surface roughness, wettability, and microbial adhesion exhibit multifactorial, interdependent behavior, reinforcing that structural modifications that improve specific properties may simultaneously influence other physicochemical and biological outcomes [50]. In the present study, the interfacial heterogeneity and incomplete nanoparticle dispersion observed in the SEM and CLSM analyses may have contributed to structural discontinuities within the polymer matrix, potentially compromising stress distribution during flexural loading. Furthermore, the 90° printing orientation may have influenced the flexural strength results due to the intrinsic anisotropy associated with layer-by-layer manufacturing [84]. Nevertheless, the findings suggest a possible balancing effect, in which improvements in surface and antibacterial properties may coexist with mechanical alterations without necessarily compromising the material’s clinical applicability.

These findings are reflected in the microhardness results, as the groups containing P-Chi exhibited lower hardness values associated with greater irregularity, particularly in G4, possibly due to nanoparticle exposure at the surface. Abushowmi et al. (2025) [61] demonstrated that nanoparticle agglomeration and inadequate dispersion may promote stress concentration points and weak interfacial zones within the resin matrix, favoring localized mechanical heterogeneity and structural discontinuities. In the present study, the incomplete dispersion previously observed in the SEM and CLSM analyses may have contributed to this behavior, potentially compromising stress distribution within the composite. Since crack propagation was not investigated, the failure mechanisms associated with interfacial heterogeneity remain only partially understood, which limits the present study. Nevertheless, previous studies involving nanoparticle-modified resins have reported alterations in surface morphology and failure behavior, suggesting that nanoparticle incorporation may influence crack propagation dynamics and stress dissipation within the polymer matrix [85]. Interestingly, although G6 exhibited flexural strength values slightly lower than those of its corresponding control group, G2 (112.43 ± 13.87 MPa vs. 119.13 ± 8.29 MPa; −5.6%), it presented markedly higher elastic modulus values (3494.95 ± 301.30 MPa vs. 3156.18 ± 306.20 MPa; +10.7%). These findings suggest that the combined effect of the LT resin matrix and higher P-Chi concentration may have increased structural rigidity without a proportional improvement in ultimate mechanical resistance, potentially reflecting alterations in stress redistribution dynamics within the composite. This behavior may be associated with localized mechanical heterogeneity promoted by nanoparticle agglomerates, which could have partially restricted polymer chain mobility and modified crack propagation pathways. In this context, heterogeneous interfacial regions may have contributed simultaneously to localized stress dissipation and structural fragility, potentially explaining the dissociation observed between elastic modulus and flexural strength.

Thus, the synergy between the physicochemical and mechanical properties promoted relevant findings regarding antibacterial performance. When antibacterial activity was assessed through diffusible substances, the absence of inhibition halos in all groups suggests that the antimicrobial effect may be mediated by alternative mechanisms rather than by molecular diffusion. This behavior is consistent with highly crosslinked polymeric systems, in which the mobility and diffusion of incorporated molecules tend to be limited, causing antimicrobial activity to depend predominantly on interfacial and surface-mediated interactions [12,27,86]. On the other hand, the reduction observed in CFU values suggests that antibacterial activity likely occurred through a contact-dependent mechanism mediated by interactions between the material surface and bacterial cells. Contact-dependent antibacterial mechanisms may involve alterations in surface polarity and electrostatic interactions at the material–bacteria interface, as described by Thongthai et al. (2021) [87], who demonstrated that positively charged quaternary ammonium groups can disrupt bacterial membranes and reduce microbial adhesion at concentrations ≥0.5 wt%. Although mediated by distinct chemical functionalities, a similar interfacial phenomenon may have occurred in the present study, since ionizable phosphate groups could modify surface polarity and electrostatic interactions at the material–bacteria interface. In agreement with Srinivasan et al. (2025) [88], phosphorylation increases the aqueous solubility of CS over a broader pH range by incorporating hydrophilic phosphate groups (–PO_4_^2−^) into the polymeric chain. In the present study, this behavior may have favored greater interfacial interaction in aqueous environments, potentially enhancing interactions with biofilm matrices in which *S. mutans* develops.

However, antimicrobial activity frequently increases with nanoparticle concentration, as demonstrated by Abozaid et al. (2025) [89], in which higher concentrations of titanium dioxide nanoparticles enhanced the antimicrobial activity of resin-based materials. Similarly, Byun et al. (2024) [90] reported improved antimicrobial performance at increased concentrations of mesoporous silica coated with cerium oxide nanoparticles. In the present study, however, no statistically significant differences were observed between the 0.25% and 0.50% concentrations (*p* = 1.000), suggesting no dose-dependent effect within the evaluated range. This behavior may indicate that increasing the P-Chi concentration did not proportionally enhance the availability of active interfacial sites, potentially due to localized agglomeration and dispersion limitations previously observed in the SEM and CLSM analyses. In highly crosslinked systems, higher nanoparticle concentrations may not necessarily translate into greater surface availability or more effective interfacial interaction with bacterial cells. Therefore, the 0.25% concentration may represent the most balanced condition, since it achieved antibacterial performance comparable to that of 0.50% while potentially minimizing structural heterogeneity within the composite.

In this context, nanoparticle dispersion within the resin matrix may produce multifactorial optical effects mediated by factors such as matrix composition, agglomerate size, and interfacial interactions. When evaluating color alterations, the resin type influenced both chromatic variation and visual perceptibility. Fidan (2022) [91] demonstrated that the main determinant of optical behavior is the refractive index mismatch between the resin matrix and incorporated nanoparticles, since this incompatibility promotes light reflection, refraction, and scattering, directly affecting translucency and overall optical appearance. Kolb et al. (2020) [92] further demonstrated that modifications in matrix chemistry alone may alter translucency through refractive index modulation, even in the absence of additional inorganic fillers. Therefore, the absence of statistically significant differences among nanoparticle-containing groups reinforces the hypothesis that color stability is more strongly associated with the intrinsic chemical composition of the resin matrix than with nanoparticle incorporation. Moreover, the consistency observed between ΔE* and ΔE_00_ findings strengthens the clinical reliability of the results, since the ST groups exhibited clinically acceptable color alterations according to the NBS scale.

Thus, P-Chi incorporation promoted simultaneous modulation of the surface, optical, mechanical, and antibacterial behavior of the 3D-printed resins, reinforcing the multifactorial nature of nanoparticle–matrix interactions in these systems. Improved antibacterial performance and increased hydrophilicity were observed, while the overall surface topography remained relatively preserved despite nanoparticle incorporation. Although reductions in flexural strength and microhardness were identified, the 0.25% concentration appeared to provide the most balanced overall performance, since it achieved antibacterial activity comparable to that of 0.50% without proportionally increasing structural heterogeneity or mechanical impairment. The findings of the present study encourage future investigations into the performance of P-Chi-modified resins under aging protocols, including water storage, thermocycling, staining exposure, and brushing abrasion, to confirm the long-term preservation of their properties. Collectively, these findings suggest that P-Chi represents a promising multifunctional strategy for the interfacial engineering of biofunctional 3D-printed provisional resins.

## 5. Conclusions

The results demonstrated that incorporating P-Chi simultaneously modulated the surface, mechanical, optical, and antibacterial properties of 3D-printed provisional resins, highlighting the multifactorial nature of matrix–nanoparticle interactions in these systems. Increased hydrophilicity and improved antibacterial performance were observed even in the absence of diffusible mechanisms, suggesting that contact-dependent interfacial interactions predominated. Although reductions in flexural strength and microhardness were observed, higher P-Chi concentrations did not yield proportional improvements in mechanical properties despite the increase in elastic modulus, suggesting that matrix–nanoparticle interactions and structural heterogeneity may have influenced the mechanical behavior of the composites. Optical alterations remained within clinically acceptable limits, suggesting that optical behavior was more strongly associated with the intrinsic composition of the resin matrix than with nanoparticle incorporation itself. Among the evaluated concentrations, 0.25% exhibited the most balanced behavior, delivering antibacterial performance comparable to that of 0.50% while incurring lower structural impairment. Collectively, these findings suggest that P-Chi represents a promising strategy for the interfacial engineering of biofunctional 3D-printed provisional resins while preserving clinically acceptable overall performance.

## Figures and Tables

**Table 1:** Surface roughness parameters of the groups obtained by profilometry (Ra, µm), expressed as mean ± standard deviation from three measurements per specimen (*n* = 30).

Group	G1	G2	G3	G4	G5	G6
Ra (µm)	0.036 ± 0.019 ^a^	0.031 ± 0.007 ^a^	0.192 ± 0.106 ^b^	0.364 ± 0.145 ^c^	0.332 ± 0.154 ^c^	0.232 ± 0.123 ^b^

Different lowercase letters indicate statistically significant differences among the group.

**Table 2:** Knoop microhardness (KHN), expressed as mean ± standard deviation from three measurements per specimen (*n* = 30).

Group	G1	G2	G3	G4	G5	G6
KHN	19.36 ± 2.06 ^a^	20.12 ± 0.94 ^a^	19.14 ± 2.48 ^ab^	14.48 ± 2.32 ^c^	19.46 ± 1.49 ^a^	20.27 ± 0.82 ^b^

Different lowercase letters indicate statistically significant differences among the groups.

**Table 3:** WCA (θ, °)**,** expressed as mean ± standard deviation from three measurements per specimen (*n* = 11).

Group	G1	G2	G3	G4	G5	G6
WCA (θ, °)	73.07 ± 10.55 ^b^	66.33 ± 4.45 ^ab^	64.48 ± 2.44 ^a^	67.81 ± 4.71 ^b^	67.08 ± 3.93 ^b^	61.18 ± 2.02 ^a^

Different lowercase letters indicate statistically significant differences among the groups.

**Table 4:** Elastic modulus and flexural strength values obtained from the three-point bending test, expressed as mean ± standard deviation (MPa), median, and 95% confidence intervals.

Groups	Elastic Modulus (MPa) Mean ± SD	Flexural Strength Mean ± SD (MPa)	Median	95% CI (MPa)
G1	2962.56 ± 278.30 ^b^	118.13 ± 8.54 ^b^	119.65	103.80–133.34
G2	3156.18 ± 306.20 ^b^	119.13 ± 8.29 ^b^	118.67	102.44–133.78
G3	2567.18 ± 315.80 ^a^	95.55 ± 7.85 ^a^	98.29	84.02–106.81
G4	2661.18 ± 503.50 ^a^	96.83 ± 12.89 ^a^	97.53	74.63–114.43
G5	3026.50 ± 256.90 ^b^	108.13 ± 11.73 ^b^	108.83	84.35–127.44
G6	3494.95 ± 301.30 ^c^	112.43 ± 13.87 ^b^	114.17	91.00–134.80

Different lowercase letters indicate statistically significant differences among the groups.

**Table 5:** Results of color variation (ΔE* and ΔE_00_), including mean values and standard deviations (SD) (*n* = 11).

System	G1–G3	G1–G5	G2–G4	G2–G6
CIE L*a*b* (ΔE*)	1.21 ± 0.67 ^a^	0.97 ± 0.16 ^a^	3.67 ± 1.02 ^b^	3.53 ± 0.26 ^b^
CIEDE 2000 (ΔE_00_)	0.82 ± 0.52 ^a^	0.65 ± 0.10 ^a^	2.96 ± 0.77 ^b^	2.94 ± 0.11 ^b^

Different lowercase letters indicate statistically significant differences among the groups.

**Table 6:** Two-way ANOVA results for Log(CFU + 1) obtained from the CFU assay.

Source	Sum of Squares	df	Mean Square	F	*p*-Value	η^2^	η^2^p	ω^2^
Resin	0.00785	1	0.00785	0.0937	0.761	0.001	0.002	−0.013
Concentration	1.51169	2	0.75585	9.0145	<0.001	0.260	0.286	0.228
Resin × Concentration	0.51081	2	0.25541	3.0461	0.057	0.088	0.119	0.058
Residuals	3.77315	45	0.08385					

**Table 7:** Bonferroni-adjusted pairwise comparisons according to P-Chi concentration.

Comparison	Mean Difference (I − J)	95% CI	*p*-Value	Cohen’s d	Magnitude
0% vs. 0.25%	0.284	0.042 to 0.525	0.016	0.95	Large
0% vs. 0.50%	0.356	0.115 to 0.598	0.002	1.24	Large
0.25% vs. 0.50%	0.072	−0.169 to 0.314	1.000	0.23	Small

## Data Availability

Data generated and analyzed during the study are available from the corresponding author upon reasonable request.

## Abbreviations

The following abbreviations are used in this manuscript:

AM	Additive manufacturing
CAD	Computer-aided design
SLA	Stereolithography
DLP	Digital Light Processing
LCD	Liquid Crystal Display
ST	Short-term
LT	Long-term
CS	Chitosan
P-Chi	Phosphorylated chitosan
SEM	Scanning Electron Microscopy
FTIR	Fourier-Transform Infrared Spectroscopy
CSLM	Confocal Laser Scanning Microscopy
KGN	Knoop microhardness
CFU	Colony-forming unit
df	Degrees of freedom
η^2^	Eta squared
η^2^p	Partial eta squared
ω^2^	Omega squared
WCA	Water Contact Angle
